# The “real life” efficacy of dupilumab is independent of initial polyp size and concomitant steroids in CRSwNP

**DOI:** 10.1186/s40463-023-00663-4

**Published:** 2023-09-06

**Authors:** Nicholas J. Campion, Jonas Brugger, Aldine Tu, Victoria Stanek, Faris F. Brkic, Tina J. Bartosik, David T. Liu, Bruna S. Hoehl, Katharina Gangl, Julia Eckl-Dorna, Sven Schneider

**Affiliations:** 1 Department of Otorhinolaryngology, Research Laboratories 8H, Medical University of Vienna, General Hospital of Vienna, Waehringer Guertel 18-20, 1090 Vienna, Austria; 2 Center for Medical Statistics, Informatics and Intelligent Systems, Medical University of Vienna, Vienna, Austria

**Keywords:** Chronic rhinosinusitis, CRSwNP, Nasal polyp, Asthma, Biological treatment

## Abstract

**Background:**

Dupilumab significantly improves symptom control in chronic rhinosinusitis with nasal polyps (CRSwNP). Patients with large polyps at the initiation of treatment (total polyp score (TPS) ≥ 5) have been the focus in published studies. Patients with significant burden of disease but small polyps (TPS ≤ 4) have not yet been evaluated for clinical response. This study set out to evaluate the benefit of dupilumab treatment on cohorts of small (TPS ≤ 4) compared to large polyps (TPS ≥ 5). Furthermore, benefit of concomitant oral and/or nasal steroid therapy has been evaluated.

**Methods:**

97 patients with CRSwNP, who were begun on dupilumab between January 2020 and October 2021, were included. All patients were followed-up for 6 months. At each visit they underwent nasal endoscopy, smell identification tests and filled out validated patient questionnaires.

**Results:**

Significant drops in TPS were seen in both patient groups after 6 months of therapy, dropping from a median score of 3 to 0 and from 6 to 2 in patients with small and large polyps respectively. Furthermore, a linear mixed model calculated a drop of 22% and 24% in TPS per month in patients with small and large polyps respectively with no significant difference in rate of decline. Finally the model showed that neither oral nor nasal steroids influenced the rate of response to dupilumab therapy.

**Conclusions:**

Polyp size at the initiation of dupilumab therapy and whether patients continue to take steroid therapy does not appear to influence effectiveness of dupilumab treatment.

**Supplementary Information:**

The online version contains supplementary material available at 10.1186/s40463-023-00663-4.

## Background

Chronic rhinosinusitis with nasal polyps (CRSwNP) is a salient subtype of chronic rhinosinusitis (CRS) and is a major health problem with an estimated prevalence in western countries of 1.9–2.7% [1, 2]. The economic burden of the disease is high with direct annual costs estimated at 5.7 billion USD in the USA [3]. The quality of life of patients with CRSwNP can be severely impaired as they struggle with symptoms of nasal obstruction, rhinorrhea,  and facial pain [4, 5].

Nasal polyps are inflammatory, nonmalignant mucosal projections which are characterized by T effector cell activation and impaired regulatory T cell function. Two major inflammatory subtypes of polyps have emerged depending on the predominance of the helper T cell subset and are thus classified as Type 2 or Type 1 predominant. Type 2 predominant polyps are characterized by a high density of eosinophil infiltrates and presence of Type 2 cytokines whereas Type 1 predominant polyps have a T helper (Th) Th1/Th17 cell bias with neutrophilic inflammation [6–8].

The majority of patients with CRSwNP are treated with local nasal steroid sprays and nasal douches, moving onto to short courses of systemic steroids and/or antibiotics if symptoms do not improve or for acute exacerbations [9, 10]. Many patients will go on to need surgery which historically has often been performed multiple times due to polyp recurrence [10]. However, within the past few years several monoclonal antibodies such as omalizumab (anti-IgE), mepolizumab (anti-interleukin (IL)-5) and dupilumab (anti-IL-4 receptor α) have gained regulatory approval and offer relief for some patients with severe CRSwNP who do not respond to standard treatment [11–13]. Due to cost, these treatments are not first line and with regards to dupilumab European guidelines were published in 2020 outlining the criteria patients need to fulfill prior to prescription. These criteria are still being fine-tuned and currently biologicals are typically reserved for patients with large polyps. Polyps can be scored according to the Gevaert endoscopic grading system, which grades polyps in each nasal cavity from 0 to 4 and totals them to give a total polyp score (TPS) of 0–8 [13]. Indeed, in the landmark LIBERTY NP trials for dupilumab only patients with TPS of 5 and above were included into the trial [14], which was of course logical as these patients do typically suffer from worse symptoms [10]. However in clinical practice patients with smaller polyps often also report high symptom burden. Appropriate patient selection for biological treatment still remains a challenging task due to high therapy costs, lack of predictive markers and patients who do not fit into current guidelines for therapy having not yet been evaluated for treatment response.

With this debate in mind, we performed a retrospective analysis of patients diagnosed with CRSwNP attending our university ear-nose-throat (ENT) clinic, who were begun on dupilumab from January 2020 until October 2021. Patients were monitored for a minimum of 6 months. We monitored the change over time in polyp size, sense of smell and quality of life (QoL) scores in patients with either small (TPS = 0–4) or large (TPS = 5–8) polyps at the initiation of dupilumab therapy to see whether this had an influence on the rate of treatment effectiveness. We also looked to see whether the number of previous surgeries, coexistent steroid use, asthma status and non-steroidal anti-inflammatory drug (NSAID) exacerbated respiratory disease (N-ERD) status had an influence on treatment response.

## Methods

### Study conduct

This retrospective study was conducted entirely at the Medical University of Vienna ENT clinic at the general hospital of Vienna in Vienna, Austria. Retrospective analysis of this patient population was approved by the Ethical Committee at the Medical University of Vienna (EK 2222/2021).

### Study population

97 patients diagnosed with CRSwNP, confirmed via history and nasal endoscopy, who were initiated on subcutaneous injection therapy with dupilumab every 2 weeks from January 2020 until October 2021 were included in this study. Patients were typically seen at baseline, and then at 1, 3 and 6 months post the initiation of dupilumab therapy.

### Criteria for dupilumab therapy and study inclusion

In Austria guidance for the prescription of Dupixent (dupilumab) is based off the European Medicines Agency (EMA) section on therapeutic indication for dupilumab in CRSwNP and the EUFOREA paper [15, 16] which state that “Dupixent is indicated as an add-on therapy with intranasal corticosteroids for the treatment of adults with severe CRSwNP for whom therapy with systemic corticosteroids and/or surgery do not provide adequate disease control”. All patients referred to our department were already on nasal steroid therapy and had either undergone previous surgery and/or treatment with oral steroids. In all cases where the patients suffered from severe uncontrolled disease [10, 15] the options, benefits and side effects of primary or revision surgery vs dupilumab were thoroughly discussed with the patients and they were allowed to come to their own fully informed decision. All patients who subsequently decided on and consented to therapy with dupilumab were advised to continue their topical nasal steroids as per current guidelines. The dose of dupilumab was 300 mg administered with a subcutaneous injection via single use pen every 2 weeks for the duration of the observational period.

### Examination procedure

At every visit, all patients underwent a complete ENT examination including inspection of the middle meatus of the nose using a standard rigid nasal endoscope. Endoscopic examination was done in a standardized manner which included assessment of polyp size through the use of the Gevaert endoscopic grading system [13], which grades polyps in each nasal cavity from 0 to 4; 0 = no visible nasal polyps, 1 = small nasal polyps in the middle meatus not reaching below the inferior border of the middle turbinate; 2 = nasal polyps reaching below the lower border of the middle turbinate; 3 = large nasal polyps reaching the lower border of the inferior turbinate or polyps medial to the middle turbinate; 4 = large nasal polyps causing complete obstruction of the inferior nasal cavity. Scores were then totaled to provide a TPS ranging from 0 to 8. Additionally at each visit, patients underwent smell assessment through the use of the German version of the Sniffin’ Sticks 16 Identification Test which asses the ability to recognize 16 odors. Patients were graded as having significant impairment of smell if they had anosmic scores on the Sniffin’ Sticks 16 Identification Test (8/16 or below) as previously defined [17]. Furthermore, they were asked to complete a questionnaire which inquired about their demographic data, asthma status, recent antibiotic and steroid use, the presence of any allergies and number of previous surgeries. They were then also asked to fill out German translations of the Sino Nasal Outcome Test 22 (SNOT-22) which assesses disease specific morbidity and has been validated for use in German speaking populations [18], the European Quality of Life Five Dimension 3 Level (EQ-5D-3L) which assesses general health related quality of Life, and the Patient Health Questionnaire 2 (PHQ-2) which screens for major depressive disorders. Patients who had coexistent asthma were also asked to complete German translations of the asthma control test (ACT) and the Mini Asthma Quality of Life Questionnaire (Mini AQLQ) which assess asthma control and disease specific burden, respectively.

### Data collection

All data was transferred onto a password protected excel file on a hospital computer in a depersonalized manner by Aldine Tu (AT), who is employed at the University clinic as a lab technician.

### Statistical analysis

Statistical analysis was done using R, version 3.6.1 and was performed by a qualified statistician working in collaboration with our department, Jonas Brugger (JB). The significance level was set to α = 0.05. Median and interquartile range of all scores were calculated at baseline and 6 months post therapy. To investigate which patients profit from dupilumab therapy, we fitted linear models for each of metric endpoints (TPS, SNOT-22, Sniffin’ Sticks, EQ-5D-3L, PHQ-2). The respective score was defined as the dependent variable, and indicator variables for a starting TPS of 0–4 or 5–8, asthma, presence of allergies, N-ERD, the number of previous surgeries for CRS and indicator variables for antibiotic- and oral steroid use as well as their interactions with time (in months) were defined as the explanatory variables. Separate models were calculated with initial TPS score and an indicator variable for nasal steroid use as explanatory variables due to the large number of missing observations of the latter. The models were also corrected for the baseline value of the respective score and an indicator variable if patients had been required to take oral steroids for their CRSwNP in the first 6 months after initiation of therapy. Additionally, a random effect was included for the patients. In case the score is expected to decrease, the model was defined using the logarithm of the respective score, since we expected the decrease of the score to be exponential. In this case 0 values were set to 0.25 in order for the logarithm to be defined. This was the case for TPS, SNOT-22, EQ-5D-3L and the PHQ-2 score. Note that the estimates in this model correspond to a relative difference over time or to a reference group. To investigate which asthmatic patients see improvement in their condition, additional linear models were fitted with the ACT score and the 4 domains of the Mini AQLQ score as the dependent variables, and the same explanatory variables as above with the exception of asthma and its interaction with time, since this score only applies for asthmatics. Again, the model was corrected for intake of oral steroids and the baseline score and a random effect for the patient was included. Estimates for all explanatory variables were calculated and confidence intervals as well as the respective *P* values were calculated for estimated changes over time. In case of the starting TPS, 0–4 was defined as the reference group, therefore estimates and confidence intervals of the group 5–8 correspond to the estimated difference to the group with starting values between 0 and 4. Due to the varying number of follow-up observations, all coefficients and confidence intervals were estimated robustly using robustlmm- package [19]. The estimated change in all scores with regards to initial TPS, asthma, allergies, N-ERD and number of previous surgeries for CRS was displayed with line charts up to a follow-up of 6 months. To display the trajectories of levels within a factor, the other factors were defined to be the most common level within the total patient collective with the exception of N-ERD. The median of two previous surgeries for CRS as an average baseline of the respective score was assumed for estimating the difference in trajectories of other factors. We added the actual trajectories of the patients used in the respective models and added jitter for better readability. No correction for multiple testing was done, therefore all *P* values are of descriptive, hypothesis-generating character.

## Results

### Overview of study population

Patients with diagnosed CRSwNP eligible for the initiation of dupilumab therapy and with no prior treatment with biologicals, attending our University clinic for ENT between January 2020 and October 2021 were included in this retrospective study. In total 97 patients were included in the analysis. 62.9% of these patients were male, the average age was 46.3 years. 64.9%, 70.1% and 51.5% suffered from asthma, allergy and N-ERD, respectively. Of the 97 patients, 90 had previously undergone surgical treatment for their CRSwNP with 7 patients not having had any previous surgery. For a detailed breakdown of patient demographics please refer to Table 1. Side effects of dupilumab were reported by 16.5% of patients. Of the reported side effects joint pain was the most common with 44%, followed by itching or rash at the injection site (25%), followed by tiredness (12.5%). All the side effects were reported as mild and none of the patients expressed the wish to stop treatment with dupilumab as a result of them.

**Table 1:** Patient demographics

	Initial TPS ≤ 4	Initial TPS ≥ 5	Total
Total [%]	61 [62.9%]	36 [37.1%]	97 [100%]
Male [%]	34 [55.7%]	27 [75%]	61 [62.9%]
Female [%]	27 [44.3%]	9 [25%]	36 [37.1%]
Age [SD]	45.7 [14.5]	47.3 [13.8]	46.3 [14.2]
YoD [IQR]	2010 [2000–2014]	2005 [1999–2013]	2010 [2000–2013]
Asthma [%]	45 [73.8%]	18 [50%]	63 [64.9%]
Allergy [%]	44 [72.1%]	24 [66.7%]	68 [70.1%]
N-ERD [%]	34 [55.7%]	16 [44.4%]	50 [51.5%]
No. ESS [IQR]	2 [1–3]	2 [1–2]	2 [1–3]

*YoD* year of diagnosis, *SD *standard deviation, *IQR* interquartile range, *N-ERD* NSAID-exacerbated respiratory disease, *ESS* endoscopic sinus surgery, *TPS* total polyp score

### Dupilumab significantly reduces objective polyp size after 6 months regardless of initial polyp score

**Fig. 1:**
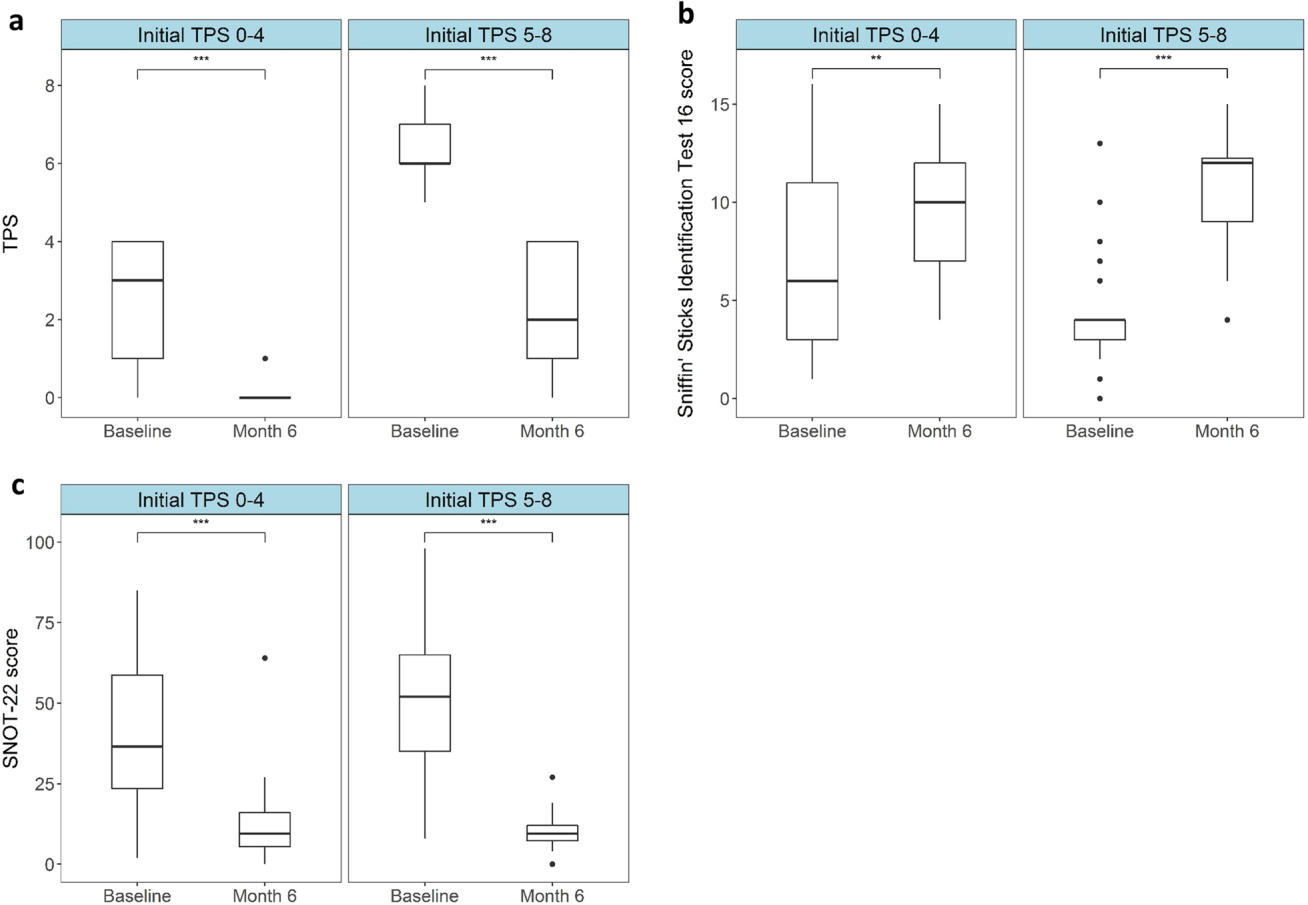
Significant improvements in TPS, SNOT-22 and smell identification are seen after 6 months of dupilumab in both ^Initial^TPS ≤ 4 and ^Initial^TPS ≥ 5 groups. Box plots indicating median total polyp score (TPS) (y-axis = score 0–8) (**a**), Sniffin’ Sticks 16 scores (y-axis = score 0–16) (**b**) and total Sino Nasal Outcome Test 22 score (SNOT-22) (y-axis = score 0–110) (**c**) at baseline and after 6 months of dupilumab therapy (x-axes). Thick lines within the box plots indicate the median, the edges indicate the lower and upper quartiles and the whiskers indicate variability outside the upper and lower quartiles. Single dots represent outliers. Significant differences are indicated by stars (**: *P* ≤ 0.01, ***: *P* ≤ 0.001)

In order to determine if the effectiveness of dupilumab was influenced by initial TPS prior to the onset of dupilumab therapy, patients were grouped into having either large (^Initial^TPS ≥ 5) or small (^Initial^TPS ≤ 4) polyps. The median TPS score was 3 (interquartile range (IQR): 1–4) and 6 (IQR: 6–7) in the ^Initial^TPS ≤ 4 and ^Initial^TPS ≥ 5 groups respectively. After 6 months of therapy the median TPS had dropped to 0 (IQR: 0–0) in the ^Initial^TPS ≤ 4 group and to 2 (IQR: 1–4) in the ^Initial^TPS ≥ 5 group. Both of these drops were statistically significant (*P* ≤ 0.001) (Fig. 1a and Table 2).

**Table 2:** Medians and interquartile ranges of assessed parameters at baseline and after 6 months of dupilumab therapy

	Initial TPS ≤ 4	Initial TPS ≥ 5	Total
*TPS*			
Baseline	3 [1–4]	6 [6–7]	4 [2–6]
Month 6	0 [0–0]	2 [1–4]	0 [0–1]
*SNOT-22*			
Baseline	36.5 [23.5–58.8]	52 [35–65]	40 [28–61.5]
Month 6	9.5 [5.5–16]	9.5 [7.3–12]	9.5 [6–14.8]
*Sniffin´ sticks*			
Baseline	6 [3–11]	4 [3–4]	4 [3–9]
Month 6	10 [7–12]	12 [9–12.3]	11 [8–12]
*EQ-5D-3L*			
Baseline	6 [5–7]	6 [5–7]	6 [5–7]
Month 6	5 [5–5]	5 [5–6]	5 [5–6]
*PHQ-2*			
Baseline	1 [0–2]	2 [0–2]	1 [0–2]
Month 6	0 [0–1]	0 [0–0]	0 [0–0.8]
*ACT*			
Baseline	23 [19–24]	19 [14.5–21]	22 [18.5–24]
Month 6	25 [24–25]	24 [22–25]	25 [23–25]
*Mini AQLQ*			
Baseline	5.9 [4.5–6.5]	4.5 [3.8–5.6]	5.6 [4.1–6.3]
Month 6	6.7 [6.5–6.9]	6.6 [5.9–6.9]	6.7 [6.4–6.9]

Numbers indicate medians and numbers in brackets indicate the interquartile ranges

*TPS* total polyp score, *SNOT-22* Sino Nasal Outcome Test 22, *EQ5D-3L* European Quality of Life Five Dimension 3 Level, *PHQ-2* Patient Health Questionnaire 2, *ACT* Asthma Control Test, *Mini AQLQ* Mini Asthma Quality of Life Questionnaire

### Objective improvement in smell identification occurs irrespective of initial polyp size and patients without previous surgery appear to have better smell function after 6 months of dupilumab

Smell ability at baseline and 6 months after dupilumab therapy was determined using the Sniffin’ Sticks 16 Identification Test which tests 16 odors. Scores are out of 16 with higher scores indicating better smell function. Median baseline smell identification scores were 6/16 (IQR: 3–11) and 4/16 (IQR: 3–4) in the ^Initial^TPS ≤ 4 and ^Initial^TPS ≥ 5 groups respectively. These scores rose to 10/16 (IQR: 7–12, *P* ≤ 0.01) and 12/16 (IQR: 9–12.3, *P* ≤ 0.001) in the ^Initial^TPS ≤ 4 and ^Initial^TPS ≥ 5 groups respectively after 6 months of dupilumab therapy (Fig. 1b and Table 2). Interestingly, when we compared patients who had not been previously operated on to those who had, those without previous surgery had significantly better olfactory function after 6 months of dupilumab than those who had undergone previous surgery (mean without surgery (13/16), mean with previous surgery (10/16), *P* value = 0.0012 according to t-test), although this finding needs to be interpreted with extreme caution due to the large imbalance in group sizes (n = 7 without previous surgery and n = 90 with previous surgery). (Additional file 1: Table E1).

### Significant improvements in patient reported outcomes are seen regardless of initial TPS score after 6 months of dupilumab therapy

At each clinical visit patients filled out patient reported outcome questionnaires to assess disease burden. Median baseline SNOT-22 scores were higher in the ^Initial^TPS ≥ 5 group at 52/110 (IQR: 35–65) than the ^Initial^TPS ≤ 4, which was at 36.5/110 (IQR: 23.5–58.8). These scores dropped significantly in both groups after 6 months of dupilumab; to 9.5 in both groups (IQR ^Initial^TPS ≤ 4: 5.5–16, IQR ^Initial^TPS ≥ 5: 7.3–12, *P* of change for both groups ≤ 0.001) (Fig. 1c and Table 2). Significant drops were also seen in all domains (as previously defined) [20] of the SNOT-22 score (Additional files 1 and 2: Table E2 and Figure E1).

**Fig. 2:**
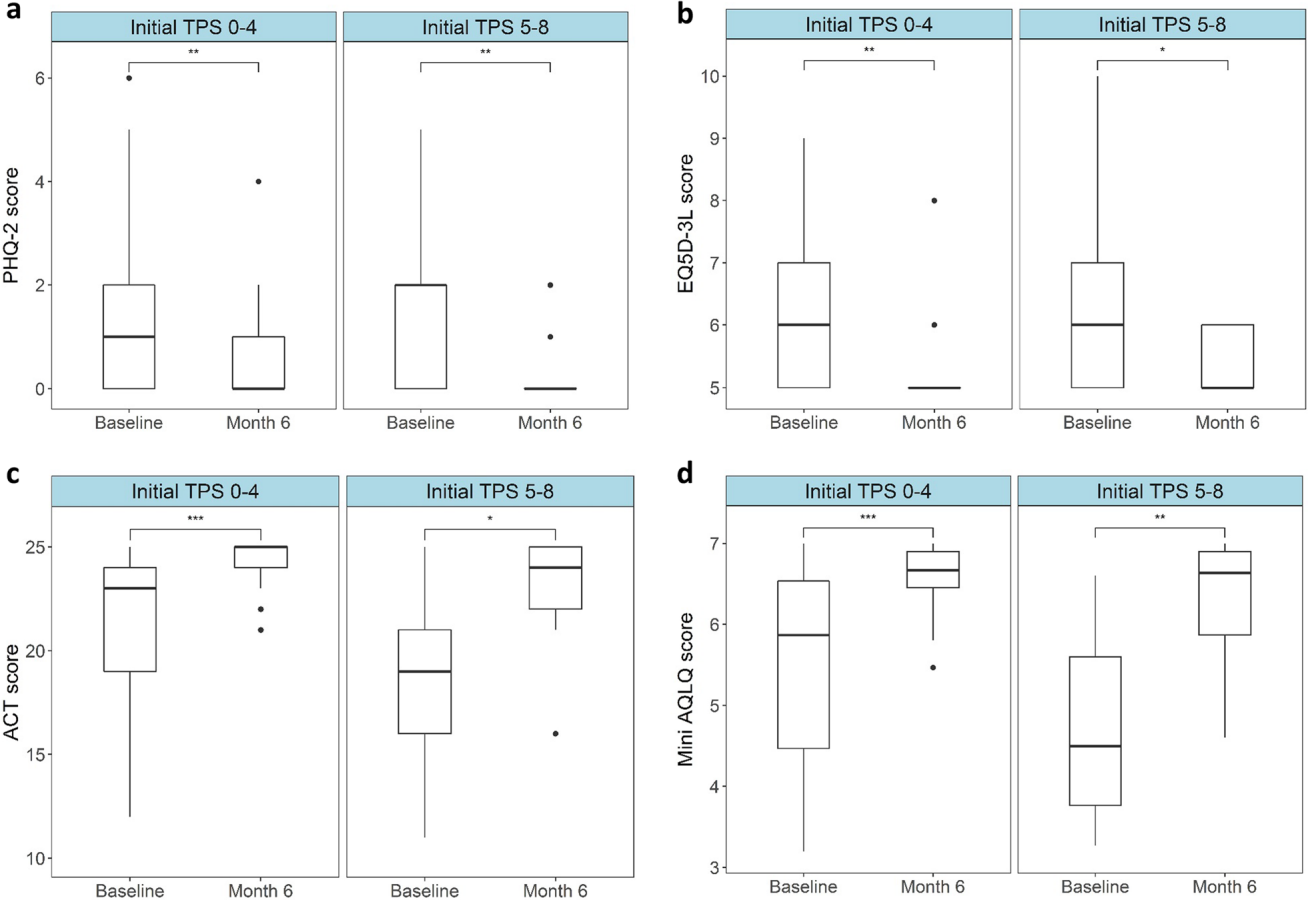
Significant improvements in patient reported outcome scores assessing general, mental and asthmatic health are seen in both ^Initial^TPS ≤ 4 and ^Initial^TPS ≥ 5 groups after 6 months of dupilumab. Box plots indicating median of Patient Health Questionnaire 2 (PHQ-2) (y-axis = score 0–6) (**a**), European Quality of Life Five Dimension 3 Level (EQ5D-3L) (y-axis = score 0–15) (**b**), Asthma Control Test (ACT) (y-axis = score 0–25) (**c**) and Mini Asthma Quality of Life Questionnaire (Mini-AQLQ) (y-axis = score 0–7) (**d**) scores at baseline and after 6 months of dupilumab therapy (x-axes). Thick lines within the box plots indicate the median, the edges indicate the lower and upper quartiles and the whiskers indicate variability outside the upper and lower quartiles. Single dots represent outliers. Significant differences are indicated by stars (*: *P* ≤ 0.05, **: *P* ≤ 0.01, ***: *P* ≤ 0.001)

Patients were also assessed on their general and mental health through the use of the EQ5D-3L and PHQ-2 scores. Both patient groups saw significant improvement in their overall mental outlook with scores dropping from 1 (IQR: 0–2) to 0 (IQR: 0–1) and 2 (IQR: 0–2) to 0 (IQR: 0–0) in PHQ-2 scores of the ^Initial^TPS ≤ 4 and ^Initial^TPS ≥ 5 groups respectively (*P* of change for both groups ≤ 0.01). Drops in the total EQ5D − 3L were small but nevertheless significant, decreasing from a median score of 6 to 5 (IQR: 5–7 for both groups at baseline, IQR: 5–5 for ^Initial^TPS ≤ 4 and IQR: 5–6 for ^Initial^TPS ≥ 5 after 6 months of dupilumab therapy) in both groups (*P* of change ≤ 0.01 for ^Initial^TPS ≤ 4 and ≤ 0.05 for ^Initial^TPS ≥ 5) (Fig. 2a, b, Table 2).

For patients with coexistent asthma (n = 63), ACT and mini AQLQ questionnaires were used to assess change in asthma burden while taking dupilumab. Both patient groups displayed better asthma control with the median ACT rising from 23/25 (IQR: 19–24) to 25/25 (IQR: 24–25) and from 19/25 (IQR: 14.5–21) to 24/25 (IQR: 22–25) in the ^Initial^TPS ≤ 4 (*P* of change ≤ 0.001) and ^Initial^TPS ≥ 5 (*P* of change ≤ 0.05) groups respectively. This was also true with regards to asthma burden with the total mini AQLQ rising from 5.9/7 (IQR: 4.5–6.5) to 6.7/7 (IQR: 6.5–6.9) and from 4.5/7 (IQR: 3.8–5.6) to 6.6/7 (IQR: 5.9–6.9) in the ^Initial^TPS ≤ 4 (*P* of change ≤ 0.001) and ^Initial^TPS ≥ 5 (*P* of change ≤ 0.01) groups, respectively (Fig. 2c, d, Table 2).

### No significant difference between the rate of polyp size decrease in patients with small and large polyps at baseline

**Fig. 3:**
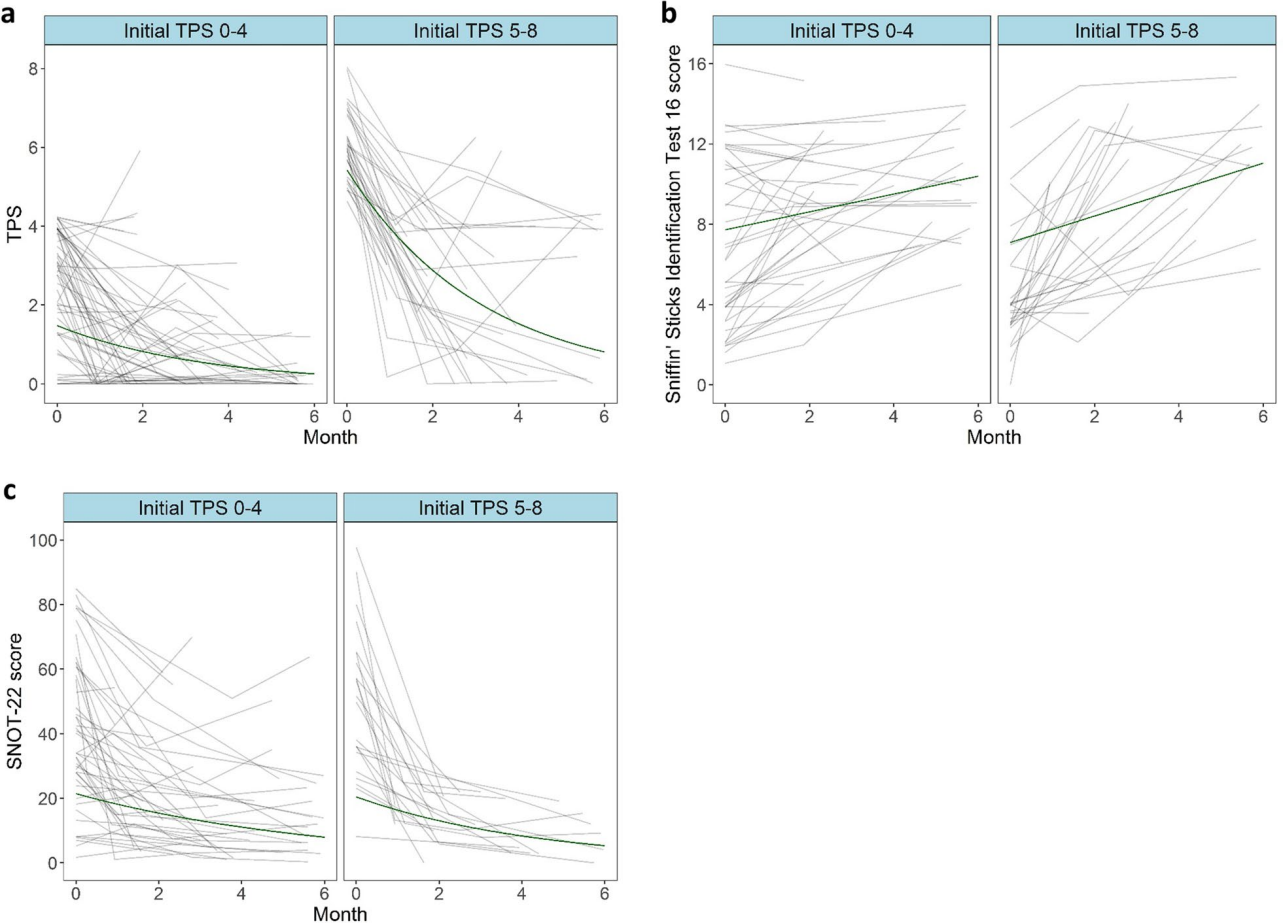
No significant differences in rate of polyp as well as symptom burden reduction and improvements in smell function are seen between ^Initial^TPS ≤ 4 and ^Initial^TPS ≥ 5 groups. Spaghetti plots produced through statistical modelling display rate of change across time (0–6 months, x-axes) in TPS (y-axis = score 0–8) (**a**), Sniffin´ Sticks 16 scores (y-axis = score 0–16) (**b**) and SNOT-22 score (y-axis = score 0–110) (**c**). Black lines represent individual patients and green lines indicate the estimated rate of change according to the statistical model

To investigate which patients benefit from dupilumab we fitted linear models for each of metric endpoints (TPS, SNOT-22, Sniffin’ Sticks 16, EQ-5D-3L, PHQ-2). The rate of change calculated by the model was compared in between ^Initial^TPS ≤ 4 and ^Initial^TPS ≥ 5 groups. The model predicted relative change of TPS size per month of 0.78 and 0.76 (22% and 24% reduction per month) for the ^Initial^TPS ≤ 4 and ^Initial^TPS ≥ 5 groups, respectively while under dupilumab therapy, these rates of decrease were not significantly different (*P* = 0.6) (Fig. 3a and Table 3). The only patient factor that influenced this rate of decline to a significant degree were patients suffering with N-ERD (n = 50), in which the relative decline of polyp size per month was slower at 0.89 and 0.87 for the ^Initial^TPS ≤ 4 and ^Initial^TPS ≥ 5 groups respectively (*P* < 0.001) (Data not shown).

**Table 3:** Calculated rates of change per month across all assessed parameters while on dupilumab according to the linear models

	Initial TPS ≤ 4	Initial TPS ≥ 5	*P* value of difference
TPS [RCpM]	0.78	0.76	0.57
SNOT-22 [RCpM]	0.88	0.83	0.07
Sniffin´ sticks [LCpM]	0.54	0.75	0.08
EQ-5D-3L [RCpM]	0.99	0.99	0.67
PHQ-2 [RCpM]	0.83	0.85	0.29
ACT [LCpM]	0.21	0.19	0.8
Mini AQLQ [LCpM]	1.59	2.26	0.13

*RCpM* relative change per month, *LCpM* linear change per month, *TPS* total polyp score, *SNOT-22* Sino Nasal Outcome Test 22, *EQ5D-3L* European Quality of Life Five Dimension 3 Level, *PHQ-2* Patient Health Questionnaire 2, *ACT* Asthma Control Test, *Mini AQLQ* Mini Asthma Quality of Life Questionnaire

### No significant differences in rates of improvement in symptom burden between patients with small and large polyps at baseline

**Fig. 4:**
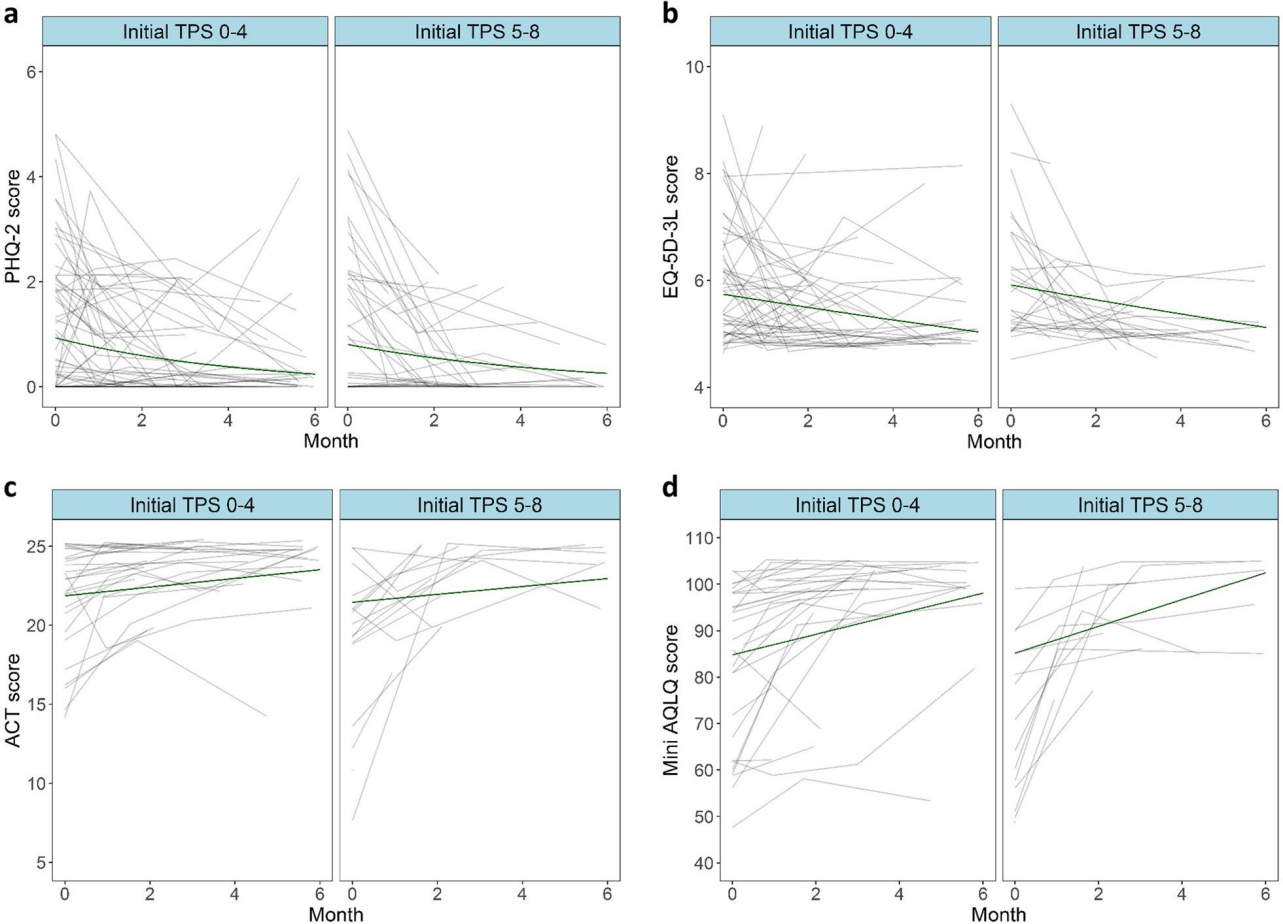
No significant differences in rate of general, mental and asthmatic health burden are seen between ^Initial^TPS ≤ 4 and ^Initial^TPS ≥ 5 groups. Spaghetti plots produced through statistical modelling display rate of change across time (0–6 months, x-axes) in PHQ-2 (y-axis = score 0–6) (**a**), EQ5D-3L (y-axis = score 0–15) (**b**), ACT (y-axis = score 0–25) (**c**) and Mini-AQLQ scores (y-axis = score 0–7) (**d**). Black lines represent individual patients and green lines indicate the estimated rate of change according to the statistical model

A minor difference in linear rate of improvement in smell identification was seen between the two groups with estimated improvement lying at + 0.54 and + 0.75 points per month for the ^Initial^TPS ≤ 4 and ^Initial^TPS ≥ 5 groups respectively, which did not reach significance *P* = 0.07 (Fig. 3b and Table 3). Additionally, minor differences in relative rates of improvement in the SNOT-22 were seen at 0.88 and 0.83 for the ^Initial^TPS ≤ 4 and ^Initial^TPS ≥ 5 groups respectively, which also did not reach statistical significance (*P* = 0.07) (Fig. 3C and Table 3). Rates of improvement per month in the EQ-5D-3L, PHQ-2, ACT and Mini-AQLQ did not differ significantly between the two groups (Fig. 4 and Table 3).

### Concurrent use of local nasal or oral steroids and/or antibiotics while taking dupilumab does not affect the rate of polyp size decline

The final analysis we performed using the statistical model was to assess if there was a synergistic effect of taking steroids and/or antibiotics while under dupilumab therapy. Coexistent nasal and/or oral steroids did not significantly alter the rate of polyp size decrease, SNOT-22 score, smell identification, EQ-5D-3L and PHQ-2 (Additional file 1: Table E3). Interestingly, the intake of oral but not nasal steroids significantly increased the rate of improvement in the ACT and Mini AQLQ by 0.84 (*P* ≤ 0.001) and 2.68 (*P* ≤ 0.01) points per month respectively (Additional file 1: Table E3). The intake of antibiotics did not influence the rate of change of polyp size, Sniffin’ Sticks 16, EQ-5D-3L, ACT or Mini AQLQ, however we did see small but significantly faster rates of improvement in the SNOT-22 (*P* ≤ 0.001) and the PHQ-2 (*P* ≤ 0.05) (Additional file 1: Table E4).

## Discussion

To the best of our knowledge, we are the first to show that in a real life setting initial polyp size and concurrent steroid use did not appear to influence the effectiveness of dupilumab treatment on CRSwNP disease specific parameters. We observed significant improvements in patients after 6 months of dupilumab in terms of polyp burden, smell identification and QoL assessed through patient reported outcome questionnaires in both patients with small and large polyps at baseline. Additionally, using a novel statistical linear model, we were able to show that the reduction of polyp size as well as improvement in quality of life measures were independent of initial polyp size. Finally, we were also able to show with the model that there was no synergistic effect on polyp size or CRSwNP disease specific burden for patients who continued to report taking steroids while on dupilumab therapy.

Since dupilumab’s approval by the FDA and EMA in 2019, numerous guidelines concerning its recommended application have been released. EPOS 2020 criteria suggest that patients should have a SNOT-22 score > 40 and be anosmic, which is typically only seen in patients with very large polyps and therefore high TPS scores [10]. Furthermore, some national guidelines have further adapted the recommendations of the EPOS 2020 and require that only patients with a TPS score ≥ 5 can be offered dupilumab, such as in Italy [14, 21]. However, based on our findings, we would argue that initiating treatment earlier could be beneficial in some patients if other standard therapies have already failed. Nevertheless, cost/benefit of dupilumab needs to be borne in mind. Studies estimating the cost efficiency of dupilumab against standard endoscopic sinus surgery (ESS) have shown that even after multiple revision surgeries, ESS is far more cost effective [22]. Additionally in patients with CRSwNP, revision surgery is only required in approximately 20% of patients and even in these patients the average length of time to revision surgery is 7.5 years but revision rates in patients with comorbidities such as asthma and N-ERD are considerably higher (approx. 30%) with shorter times to revision surgery [23]. However, time to revision surgery is not the same as being symptom free with up to 40% of patients showing recurrence of disease already 1 year after surgery [24, 25]. Furthermore, ESS has not been shown to have a significant benefit on restoration of smell function [26], which can be hugely burdensome for some patients [27]. Furthermore, complications rates of ESS for CRSwNP are low but are nevertheless present and can result in severe patient morbidity and rarely mortality [28]. Equally however, side effects of dupilumab do also occur and include symptoms such as arthralgia and conjunctivitis, which can have significant impacts on quality of life [14].

Another important consideration is the healthcare infrastructure and geographical location of the patients. The differing prevalence of Type 2 inflammation across the globe needs to be taken into account when considering patients for biological therapy [29]. Additionally, healthcare system infrastructure influences appropriateness of some therapies. For example societal guidelines on the prescription of dupilumab differ in Europe and North America with the ICAR-RS-2021 recommending biological therapy only after previous ESS, whereas the EPOS 2020 and more recent EUFOREA guidelines have a more liberal approach with the latter having no absolute requirement for previous ESS [10, 15, 30]. As data from North America has shown that ESS is vastly more cost effective than dupilumab at current pricing [31], it can be argued from an economic perspective prescription of dupilumab before surgery is perhaps unjustified, especially as the long term immunological effects of the treatment in CRSwNP are unknown. However, dupilumab and other biologics have been used very successfully and safely in the context of allergic asthma and with randomised control trials showing clear benefits of biologics in patients with severe disease [14]. Prospective studies comparing biologics to surgery in terms of outcome and side effect profile are needed to answer the question of when is the optimal time point to introduce them. Until these are available, whether to introduce them before or after surgery will remain a hotly debated topic.

Therefore, even though dupilumab is equally effective in patients with small vs large polyps, proper informed consent of the risk, benefits and outcomes of surgery vs biological therapy in light of current healthcare costs need to be discussed carefully with patients to allow them to come to their own informed decision.

Our findings in our cohort overall are similar to previous clinical trial and “real life” data on patient response to dupilumab after 6 months of treatment in terms direct measures of nasal function i.e. TPS, SNOT-22, smell identification [14, 21, 32–34] as well as patient reported outcomes assessing general, asthmatic and mental health [33, 35–38]. These findings validate that our patient cohort is representative of the typical patients seen to benefit from dupilumab therapy in the literature. As far as we could find, no study to date has investigated response rates between patients with small and large polyps at baseline. Across all parameters we assessed, we saw significant improvements in both ^Initial^TPS ≤ 4 and ^Initial^TPS ≥ 5 groups after 6 months of dupilumab treatment. Furthermore, through the use of a linear statistical model the reduction of TPS occurred at a similar rate of approximately 25% per month in both ^Initial^TPS ≤ 4 and ^Initial^TPS ≥ 5 groups. Although no study to date has used such modelling, the gradient of TPS decline in the landmark Liberty-52 trial in the first month was approximately -0.25 and in an Italian study was -0.23, which is line with our findings [14, 21]. Only in patients with N-ERD did we see a slower reduction of polyp size. This finding is unsurprising, as many studies have showed that patients with N-ERD suffer from more severe and more treatment resistant nasal polyps [39, 40]. Considering that our population is based in Europe and the majority of nasal polyps in this region are known to be Type two dominant, we find it not surprising that the rates of polyp size reduction are no different based on initial polyp size as both patient groups in a European setting most likely represent the same pathophysiological process, just at different stages [6–8]. Based on these facts and findings we would suggest that stratifying access to dupilumab treatment based on current polyp burden is not necessary and instead, focus should be on symptom burden and the history of disease (e.g. significant symptom burden despite previous surgery and/or oral steroid use).

The final aspect we considered in this study was to see if there was any synergistic effect on patients who continued to take nasal steroids and/or antibiotics while they were under dupilumab treatment. Previous studies have shown that dupilumab therapy reduces the need for intranasal corticosteroids and antibiotic intake [12, 14, 41]. We found no synergistic effect of concomitant steroid use (oral and/or nasal) on polyp size, symptom burden or olfactory function. Only in patients with concomitant asthma did we observe a significantly faster improvement rate of Asthma burden while taking oral but not nasal steroids, which was not seen in CRSwNP disease parameters. This suggests that when patients start dupilumab therapy they could possibly be offered a trial of being weaned off their steroid therapies if they do not suffer from co-existent asthma. This is in line with current guidelines as a criteria of dupilumab effectiveness when assessing if patients should continue therapy according to the EPOS 2020 guidelines is reduced systemic steroid use [10].

Limitations of our study include the fact that it was performed at a single center and that our center is a tertiary referral center for rhinosinusitis patients across the whole of Eastern Austria. This therefore means that we tend to see CRS patients with more severe forms of the disease, as reflected by our high proportion of patients suffering from N-ERD. This may have led to selection bias. However, currently dupilumab is only indicated across Europe and America for patients with treatment resistant disease and therefore, our population is likely to be representative of the current patient group being offered dupilumab. Additionally, our study is retrospective in design and thus suffers from the fact that some patient data was missing. Our statistical model however, was adjusted for these factors and patients who did not attend their follow-up visits were assumed to be non-responders during the design of this model.

While our findings suggest that initial polyp size has no influence on patient response to dupilumab, further prospective studies are needed to confirm this and should also include an economic aspect to see whether the cost of the therapy is offset by indirect savings through factors such as fewer work days missed. Additionally, in terms of synergistic effects of concomitant nasal/systemic and/or antibiotic use, prospective studies comparing dupilumab alone vs dupilumab with steroid and or antibiotic treatment when indicated are necessary before definitive recommendations can be made.

## Conclusions

In summary, we found that patients with small or large polyps benefitted equally and showed no difference in rate of decline in total polyp score, improvement of smell function or reduction in symptom burden. We also showed no additional benefit for patients who continued with standard therapies for CRSwNP while taking dupilumab. Our results suggest that patients who suffer from uncontrolled symptoms despite standard treatment (consistent nasal steroid use as well as previous therapy with oral steroids and/or surgery), dupilumab therapy should be considered regardless of initial polyp size.

### Supplementary Information

**Additional file 1.** Supplementary tables E1-4.

**Additional file 2.** Supplementary Figure E1.

## Data Availability

The data that support the findings of this study are archived at the Medical University of Vienna but restrictions apply to the availability of these data and so they are not publicly available. Data may however be made available at reasonable request to the authors and only with permission of the Medical University of Vienna.

## Abbreviations

ACT: Asthma control test
CRS: Chronic rhinosinusitis
CRSwNP: Chronic rhinosinusitis with nasal polyps
ENT: Ear, nose, throat
EPOS: European Position Paper on Rhinosinusitis and Nasal Polyps
EQ5D-3L: European Quality of Life Five Dimension 3 Level
IL: Interleukin
IQR: Interquartile range
Mini-AQLQ: Mini Asthma Quality of Life Questionnaire
N-ERD: NSAID-exacerbated respiratory disease
NSAID: Non-steroidal anti-inflammatory drugs
PHQ-2: Patient Health Questionnaire 2
QoL: Quality of life
SD: Standard deviation
SNOT-22: Sino Nasal Outcome Test 22
Th: T-helper cell
TPS: Total polyp score
