# Supplementary material for: The “real life” efficacy of dupilumab is independent of initial polyp size and concomitant steroids in CRSwNP
Source: J Otolaryngol Head Neck Surg. 2023 Sep 6;52:56. doi: 10.1186/s40463-023-00663-4 (PMC10481502; doi:10.1186/s40463-023-00663-4)
Supplement: Supplementary file 1 — Additional file 1. Supplementary tables E1-4. [file 40463_2023_663_MOESM1_ESM.docx]

**Supplementary Information: Tables**

**Table E1: Comparison of difference in outcome after 6 months of dupilumab in previously operated and surgically niave patients:** Numbers indicate means. TPS = Total polyp score, SNOT-22 = Sino Nasal Outcome Test 22, EQ5D-3L = European Quality of Life Five Dimension 3 Level, PHQ-2 = Patient Health Questionnaire 2, ACT = Asthma Control Test, Mini AQLQ = Mini Asthma Quality of Life Questionnaire.

|  | **Mean: No Previous Surgery** | **Mean: Previous Surgery** | **p-value** |
| --- | --- | --- | --- |
| **TPS** |  |  |  |
| Baseline | 5.1 | 3.8 | 0.0975 |
| Month 6 | 0.4 | 0.9 | 0.1420 |
| **SNOT-22** |  |  |  |
| Baseline | 64.0 | 41.6 | 0.0861 |
| Month 6 | 7.8 | 12.4 | 0.2485 |
| **Sniffin´ Sticks** |  |  |  |
| Baseline | 4.8 | 5.9 | 0.6192 |
| Month 6 | 13.0 | 9.9 | 0.0012 |
| **EQ-5D-3L** |  |  |  |
| Baseline | 6.2 | 6.0 | 0.7995 |
| Month 6 | 5.4 | 5.3 | 0.7186 |
| **PHQ−2** |  |  |  |
| Baseline | 2.6 | 1.5 | 0.1205 |
| Month 6 | 0.0 | 0.5 | 0.0012 |
| **ACT** |  |  |  |
| Baseline | 19.8 | 20.6 | 0.4213 |
| Month 6 | 24.7 | 23.7 | 0.0921 |
| **Mini AQLQ** |  |  |  |
| Baseline | 5.2 | 5.3 | 0.7680 |
| Month 6 | 6.8 | 6.5 | 0.0370 |

**Table E2: Medians and Interquartile ranges of Sino-Nasal Outcome Test 22 domains at baseline and after 6 months of dupilumab therapy.** Numbers indicate medians and numbers in brackets indicate the interquartile ranges. TPS = Total polyp score.

|  | **Initial TPS ≤ 4** | **Initial TPS ≥ 5** | **Total** |
| --- | --- | --- | --- |
| **Sleep Domain** |  |  |  |
| Baseline | 12 [5-22] | 18 [10-26] | 13 [5·3-25] |
| Month 6 | 2 [0-6] | 2 [0-8] | 2 [0-6·8] |
| **Nasal Domain** |  |  |  |
| Baseline | 20 [11·5-26] | 25 [18·5-29·5] | 22 [13·3-27] |
| Month 6 | 7 [3-8·8] | 5 [3·5-7·5] | 7 [3-8] |
| **Otology Domain** |  |  |  |
| Baseline | 3 [1-7·8] | 4 [2-8] | 4 [1-8] |
| Month 6 | 1 [0-2] | 0·5 [0-2] | 1 [0-2] |
| **Emotional Domain** |  |  |  |
| Baseline | 1 [0-4] | 3·5 [1-5·8] | 2 [1-5] |
| Month 6 | 0 [0-0] | 0 [0-1] | 0 [0-0·5] |

**Table E3: Effect of Steroid use on rate of change across all assessed parameters.** RM = relative multiplier, LC = linear change. TPS = Total polyp score, SNOT-22 = Sino Nasal Outcome Test 22, EQ5D-3L = European Quality of Life Five Dimension 3 Level, PHQ-2 = Patient Health Questionnaire 2, ACT = Asthma Control Test, Mini AQLQ = Mini Asthma Quality of Life Questionnaire

|  | **Oral Steroid Use** | **P value of difference** | **Nasal Steroid Use** | **P value of difference** |
| --- | --- | --- | --- | --- |
| **TPS [RM]** | 1·00 | 0·982 | 0·96 | 0·39 |
| **SNOT-22 [RM]** | 1·07 | 0·192 | 1·03 | 0·369 |
| **Sniffin Sticks [LC]** | -0·20 | 0·299 | 0·03 | 0·820 |
| **EQ-5D-3L [RM]** | 1·00 | 0·91 | 1·00 | 0·98 |
| **PHQ-2 [RM]** | 1·01 | 0·91 | 0·95 | 0·2 |
| **ACT [LC]** | **0**·**84** | **<0**·**001** | 0·09 | 0·6 |
| **Mini AQLQ [LC]** | **2**·**68** | **0**·**01** | 0·47 | 0·54 |

**Table E4: Effect of Antibiotic use on rate of change across all assessed parameters.** RM = relative multiplier, LC = linear change. TPS = Total polyp score, SNOT-22 = Sino Nasal Outcome Test 22, EQ5D-3L = European Quality of Life Five Dimension 3 Level, PHQ-2 = Patient Health Questionnaire 2, ACT = Asthma Control Test, Mini AQLQ = Mini Asthma Quality of Life Questionnaire

|  | **Antibiotic Use** | **P value of difference** |
| --- | --- | --- |
| **TPS [RM]** | 0·87 | 0·126 |
| **SNOT-22 [RM]** | **0**·**71** | **<0**·**001** |
| **Sniffin Sticks [LC]** | 0·03 | 0·927 |
| **EQ-5D-3L [RM]** | 0·99 | 0·38 |
| **PHQ-2 [RM]** | 0·83 | 0·02 |
| **ACT [LC]** | -0·13 | 0·5 |
| **Mini AQLQ [LC]** | -0·05 | 0·95 |

**Supplementary Information: Figure Legends**

**Figure E1: Significant improvements in all domains of the SNOT-22 questionnaire are seen after 6 months of dupilumab in both ^Initial^TPS ≤ 4 and ^Initial^TPS ≥ 5 groups.** Box plots indicating median of Sleep (y-axis = score 0-40), Nasal (y-axis = score 0-40), Otology (y-axis = score 0-20) and Emotional (y-axis = score 0-10) Domains at baseline and after 6 months of dupilumab therapy (x-axes). Thick lines within the box plots indicate the median, the edges indicate the lower and upper quartiles and the whiskers indicate variability outside the upper and lower quartiles. Single dots represent outliers. Significant differences are indicated by stars (*: p≤0·05, **: p≤0·01, ***: p≤0·001).
