# Supplementary figures and images for: The “real life” efficacy of dupilumab is independent of initial polyp size and concomitant steroids in CRSwNP
Source: J Otolaryngol Head Neck Surg. 2023 Sep 6;52:56. doi: 10.1186/s40463-023-00663-4 (PMC10481502; doi:10.1186/s40463-023-00663-4)

## Slide 1
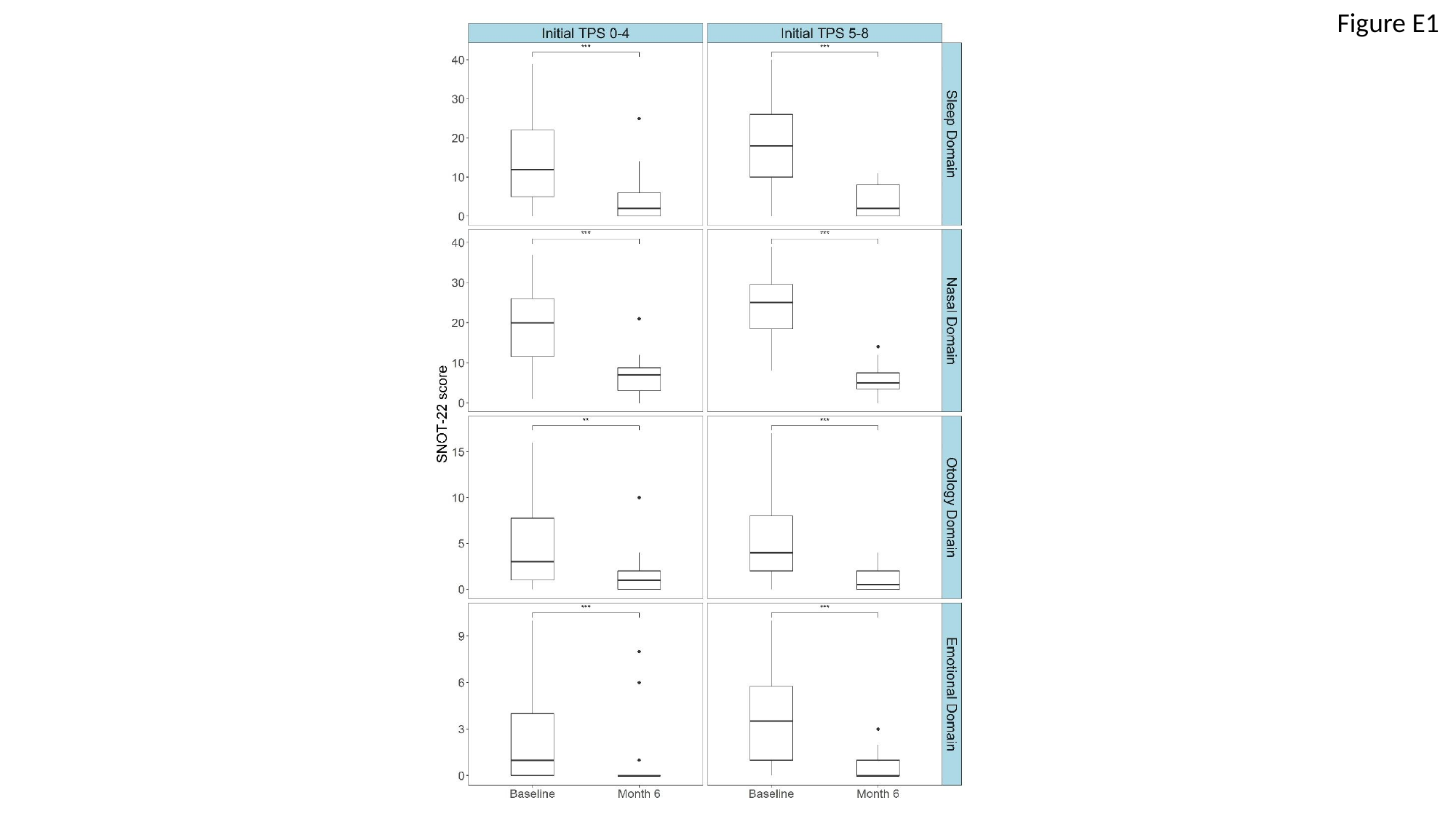

Figure E1

Supplement: Supplementary file 2 — Additional file 2. Supplementary Figure E1. [file 40463_2023_663_MOESM2_ESM.pptx]
